# Prevalence of antipsychotic use and associated adverse effects in moroccan with mental health problems

**DOI:** 10.1192/j.eurpsy.2023.1315

**Published:** 2023-07-19

**Authors:** N. El Moussaoui, A. Tounsi, S. Bahetta, S. Belbachir, A. Ouanass

**Affiliations:** Psychiatry, Arrazi Hospital of Sale, Salé, Morocco

## Abstract

**Introduction:**

Antipsychotic drugs are widely prescribed for schizophrenia and other mental disorders. They are critical in the pharmacological management of severe psychotic disorder.

The adverse effects of antipsychotics are common, with a potential negative impact on adherence and engagement. Despite this, the scientific study of the prevalence of adverse antipsychotic effects is a neglected area.

**Objectives:**

We aim to identify the prevalence of nine clinically important categories of antipsychotic adverse effects, namely: extrapyramidal symptoms; sedation; weight gain; type II diabetes; hyperprolactinaemia; metabolic syndrome, dyslipidaemia; sexual dysfunction; and cardiovascular effects

**Methods:**

This is a prospective, observational, cross-sectional study, carried out in Ar-razi hospital in Salé evaluating side effects in patients hospitalized and treated with antipsychotics within 3 months.

**Results:**

In total, antipsychotic polypharmacy was associated with increased frequency of adverse effects, and a longer duration of treatment is associated with greater severity; clozapine was more strongly associated with metabolic disturbance than other antipsychotics in three studies and olanzapine was associated with the most weight gain in three studies; hyperprolactinemia was more common in women than men, but more men noted sexual dysfunction than women;

**Conclusions:**

Antipsychotic adverse effects are diverse and frequently experienced, but are not often systematically assessed. There is a need for further scientific study concerning the management of these side effects.

**Disclosure of Interest:**

None Declared

